# Post-traumatic Delayed Bilateral Vocal Cord Paralysis Caused by Cervical Osteophytes: A Case Report

**DOI:** 10.7759/cureus.64189

**Published:** 2024-07-09

**Authors:** Takuya Yamaguchi, Hideya Itagaki, Tomoyuki Endo

**Affiliations:** 1 Emergency and Disaster Medicine, Tohoku Medical and Pharmaceutical University Hospital, Miyagi, JPN

**Keywords:** multiple head injury, airway emergency, osteophytes, bilateral vocal cord paresis, vocal cord paralyses

## Abstract

Vocal fold paralysis occurs when the function of the vagus nerve or its distal branch, the recurrent laryngeal nerve, is diminished or absent. Bilateral vocal fold paralysis can present with varying degrees of severity and is sometimes fatal. Cervical osteophytes are a rare cause of bilateral vocal fold paralysis, with only a few cases reported. A 68-year-old man was brought to the emergency department because of a disturbance in consciousness following a fall. A CT scan of the head showed multiple cranium and brain injuries, and the patient was treated conservatively by neurosurgery. The day after the injury, dysphagia and dysarthria appeared. On the third day of admission, both vocal cords were fixed bilaterally in the paramedian position, and the patient was nearly choking on sputum. A CT scan showed that the intracranial lesions gradually improved, but the vocal cord paralysis remained. A cervical CT scan was performed to investigate the cause of the vocal cord paralysis, which revealed that cervical vertebral osteophytes were compressing the tracheoesophageal groove and the glottis. The patient was transferred to the hospital for rehabilitation, although bilateral vocal cord paralysis remained. Although rare, clinicians need to be aware that cervical osteophytes can cause vocal fold paralysis, which may be manifested when combined with further trauma. It is also important to note that traumatic vocal cord paralysis can be delayed.

## Introduction

Vocal cord paralysis is defined as decreased or absent function of the vagus nerve or its distal branch, the recurrent laryngeal nerve [1]. Depending on the location of the paralysis, bilateral vocal cord paralysis may present with varying degrees of dyspnea, inspiratory stridor, aphonia, dysphagia, and aspiration. It may be fatal or require emergency airway management [1,2]. Bilateral vocal cord paralysis occurs in about 10% of all vocal cord paralyses. Trauma is reported to be present in 6.2% of bilateral vocal cord paralysis cases and 1.5% of all vocal cord paralysis cases [3,4]. Only a few cases have been reported where cervical vertebral osteophytes were the cause of vocal cord paralysis [5-7]. In this study, we encountered a case in which a cervical osteophyte was thought to have injured the tissues surrounding the recurrent laryngeal nerve due to cervical flexion caused by traumatic injury. We report the case with a literature review.

## Case presentation

A 68-year-old man came to the emergency room because of loss of consciousness after a fall from a high place. On the day of his arrival, he was pruning his garden using a stepladder when he fell and was found unconscious by a neighbor, who brought him to our hospital. His medical history included hypertension, and he was taking valsartan, bisoprolol, and benidipine. Vital signs at admission were as follows: Glasgow Coma Scale: E4V5M6, blood pressure of 189/131 mmHg, pulse of 120 beats/minute, body temperature of 36.0℃, respiratory rate of 16 breaths/minute, and oxygen saturation of 98%. He complained of neck pain. Physical examination revealed a hematoma in the left posterior neck region, but there was no tenderness in the cervical spine and no numbness or motor impairment of the extremities. Since it was unclear whether the patient fell after losing consciousness or whether he lost consciousness due to head trauma, a thorough examination was conducted. Blood tests revealed a mild inflammatory reaction but no anemia or electrolyte abnormalities (Table 1).

**Table 1:** Complete blood count data, biochemistry data, and blood gas analysis test at the time of visit WBC, white blood cell; RBC, red blood cell; Hb, hemoglobin; Hct, hematocrit; Plat, platelet; MCV, mean corpuscular volume; MCH, mean corpuscular hemoglobin; Fib, fibrinogen; APTT, activated partial thromboplastin time; PT-INR, prothrombin time-international normalized ratio; TP, total protein; Alb, albumin; T-bil, total bilirubin; AST, aspartate aminotransferase; ALT, alanine aminotransferase; ALP, alkaline phosphatase; γGT, γ-glutamyl transpeptidase; LDH, lactate dehydrogenase; BUN, blood urea nitrogen; Cre, creatinine; Na, sodium; K, potassium; Cl, chlorine; Ca, calcium; Mg, magnesium; CK, creatine kinase; CK-MB, creatine kinase-myoglobin binding; Tropnin T, cardiac muscle troponin T; CRP, C-reactive protein; PCT, procalcitonin; IL-6,interleukin-6; BE, base excess; Lac, lactate.

Parameter	Result	Reference value
Complete blood count data		
WBC	14.8	3.3–8.6 (×10^3^/μL)
RBC	4.51	4.35–5.55 (×10^6^/μL)
Hb	16.4	13.7–16.8 (g/dL)
Hct	50.2	40.7–50.1 (%)
Plat	196	158–348 (×10^3^/μL)
MCV	111.4	83.6–98.2 (fL)
MCH	36.4	30.5–34.2 (pg)
Fib	607	200–400 (mg/dL)
APTT	33.8	24–39 (sec)
PT–INR	1.08	0.90–1.15
D–dimer	69.85	0–1.0 (μg/mL)
Biochemistry data		
TP	7.1	6.6–8.1 (g/dL)
Alb	4.2	4.1–5.1 (g/dL)
T–bil	0.82	0.4–1.5 (mg/dL)
AST	36	13–30 (U/L)
ALT	20	10–42 (U/L)
ALP	253	38–113 (U/L)
γGT	169	13–64 (U/L)
LDH	367	124–222 (U/L)
BUN	17	8–20 (mg/dL)
Cre	0.86	0.65–1.07 (mg/dL)
Na	140	138–145 (mmol/L)
K	3.8	3.6–4.8 (mmol/L)
Cl	101	101–108 (mmol/L)
Ca	9.1	8.8–10.1 (mg/dL)
BS	132	70–110 (mg/dL)
CRP	3.54	0.0–0.14 (mg/dL)
PCT	0.04	0.0–0.05 (ng/mL)
IL–6	32.4	0.0–7.0 (pg/mL)
Blood gas analysis		
pH	7.337	7.35–7.450
PaO_2_	22.5	80–100 (mmHg)
PaCO_2_	54.1	35–45 (mmHg)
HCO_3-_	29	22–26 (mmol/L)
cLac	2.3	0.26~1.39 (mmol/L)

**Figure 1:**
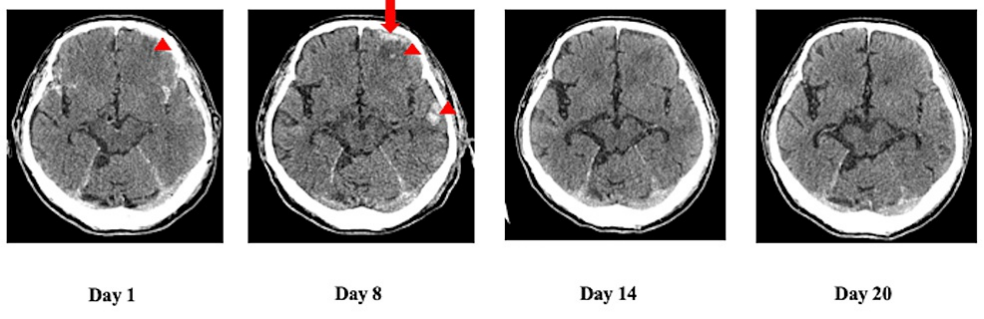
Time course of intracranial lesions Subcutaneous and epidural hematoma in the occipital regions (red arrow) and subdural hematoma in the left frontal lobe (red arrowhead). The CT scan on day 8 revealed a temporary cerebral contusion in the left frontal and temporal lobes, but the CT scan on days 14 and 20 was almost normal.

An electrocardiogram showed atrial fibrillation with a heart rate of 120 beats per minute. Echocardiography revealed an ejection fraction (EF) of 48.5% and only mild wall motion loss in the ventricular septum. A CT scan revealed a subcutaneous hematoma and skull fracture in the left temporal and occipital regions, an acute subdural hematoma in the left frontal lobe, traumatic subdural hemorrhage in the bilateral Sylvian fissures, and cerebral contusion in the left frontal and temporal lobes, but no obvious vertebral or hyoid fractures (Figure 1).

Although the possibility of a fall due to cardiogenic syncope could not be ruled out, the patient was admitted to neurosurgery because of multiple cranium and brain injuries. After neurosurgical admission, the patient was treated with intravenous antihypertensive drugs (nicardipine 1 μg/kg/min) and started on antiepileptic drugs (lacosamide 200 mg/day) to prevent symptomatic epilepsy. A CT scan was performed on the morning of the second day of admission, and although there was no evidence of cerebral contusion, dysphagia and dysarthria appeared by noon. On the third day of admission, the patient was nearly choking on sputum, and the otolaryngologist examined him and found that his vocal cords were fixed bilaterally in the paramedian position, leading to the diagnosis of bilateral vocal cord paralysis. Because of the possibility of airway emergencies, tracheal intubation was performed on the same day, and a tracheostomy was performed on the seventh day of admission.

**Figure 2:**
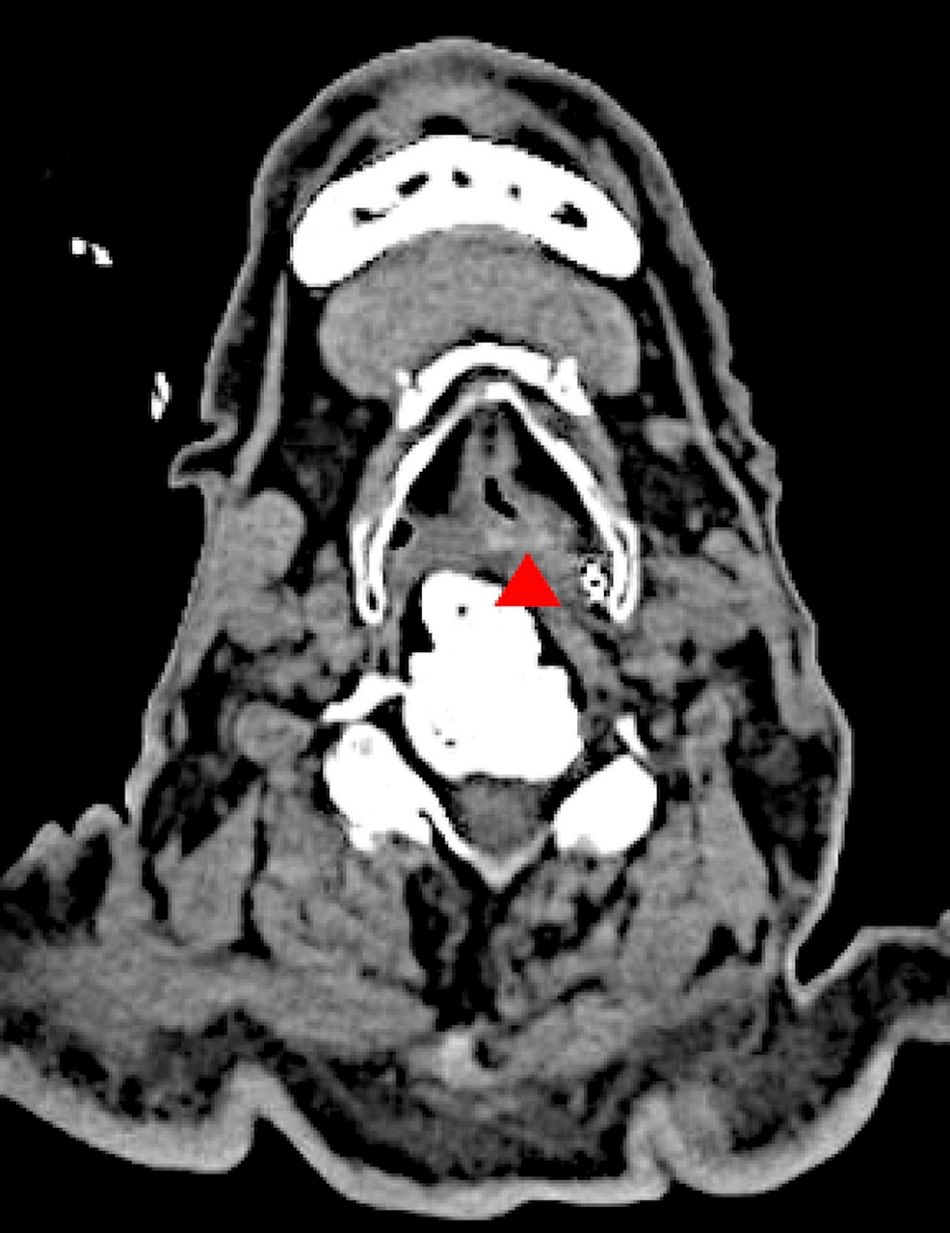
CT scan of the neck Cervical osteophytes (red arrow) compress the tracheoesophageal groove and glottis.

CT scans for head follow-up were performed on the 8th, 14th, and 20th days of admission, and the acute subdural hematoma in the left frontal lobe area and cerebral contusions in the frontal and temporal lobes gradually improved (Figure 1). Later, the otorhinolaryngologist examined the patient and found that vocal cord paralysis remained, and the patient still had pharyngeal hypoesthesia and dysphagia. On day 57 of hospitalization, an MRI scan was performed to investigate the cause of the paralysis, but no obvious cause of vocal cord paralysis was noted. On the 69th day of hospitalization, a CT scan of the cervical region revealed that the cervical vertebral osteophytes were compressing the tracheoesophageal groove, and the glottis was also compressed (Figure 2).

Based on the results of this examination, we suspected vocal cord paralysis due to recurrent nerve injury caused by the osteophytes. When we asked the patient about his history before admission, he mentioned that he had been aware of difficulty swallowing for some time. Although he still had bilateral vocal cord paralysis, his general condition was stable, and he was transferred to the hospital for rehabilitation on the 71st day of admission.

## Discussion

In this case, a patient with bilateral vocal cord paralysis who was able to speak at the time of presentation developed dysphagia and dysarthria the following day, requiring urgent airway management. We learned the following lessons from this case: bilateral vocal cord paralysis can be caused by recurrent nerve injury due to cervical osteophytes; posttraumatic vocal cord paralysis can occur later, rather than immediately after injury; and bilateral vocal cord paralysis can result from traumatic injury.

The recurrent laryngeal nerve recurs through the aortic arch on the left side and the right subclavian artery on the right side, travels through the tracheoesophageal groove, and enters the larynx behind the cricothyroid joint [8]. Therefore, the recurrent laryngeal nerve is injured when the aortic arch region, right subclavian artery region, and tracheoesophageal groove are wounded [3]. The resulting inability of the vocal folds to abduct/adduct is usually called vocal fold paralysis. Bilateral vocal fold paralysis accounts for one-third of all vocal fold paralyses and presents with a variety of symptoms, including dyspnea, stridor, dysphagia, and aspiration [2]. The most common cause of vocal cord paralysis is postoperative; other causes include malignancy, central nervous system lesions, inflammation, and trauma. In the present case, trauma was one of the causes, which can result in both bilateral and unilateral vocal cord paralysis [3,4,9,10].

The details of trauma and vocal cord paralysis are not well documented. However, a review of articles from 1955 to 1999 found a 13% incidence of bilateral vocal cord paralysis due to trauma, and in 1182 cases of bilateral vocal cord paralysis in 2024, there was a 3% (36/1182) rate of bilateral vocal cord paralysis due to trauma [4,11]. Most of the previously reported cases of traumatic bilateral vocal cord paralysis were accompanied by hyoid, skull base, or vertebral fractures, and the cases were subjected to strong directional forces to the neck or downward intracranial shearing forces, which may have resulted in damage to the recurrent nerve (Table 2) [2,12-19].

**Table 2:** Reported cases of post-traumatic triggered vocal cord paralysis

Author	Sex	Age	Type of trauma	Symptoms at the time of visit	Presence of bone fractures	Time to onset of dyspnea (days)	Treatment: tracheostomy	Outcome: improved vocal cord paralysis
Ehret [3]	M	80	Falling down	−	Yes	30	Yes	Yes
Helliwell [11]	W	65	Traffic accidents	Neck and chest pain and diplopia	No	0	Yes	No
Yu Asami [12]	M	72	Falling down	Quadriplegia	No	15	Yes	No
Yoo [13]	M	57	Falling down	Headache	Yes	1	No	No
Levine [14]	M	26	Traffic accidents	Respiratory distress	Yes	0	Yes	Yes
Latoo [15]	M	35	Strangulated the neck of the patient	Respiratory distress	Yes	0	Yes	Yes
Kunii [16]	M	39	Suicide by hanging	Coma	Yes	0	Yes	Yes
Tomar [17]	W	25	Traffic accidents	Disturbance of consciousness	Yes	0	Yes	−
Our hospital	M	68	Falling down	Disappearance of consciousness, and neck pain	Yes	1	Yes	No

In some of these reports, as in the present case, osteophytes of the cervical spine are thought to be involved [15].

Osteophytes are abnormal bone growths, bony projections forming along joints [6]. Osteophytes form at repeated stress and inflammation sites due to increased bone turnover, remodeling, and calcification with subsequent bone deposition [6]. The association between cervical osteophytes and vocal cord paralysis is supported by Yoskovitch et al. [5]. Yoskovitch et al. reported a case in which a cervical osteophyte compressed the tracheoesophageal groove and caused recurrent nerve palsy, and in this report, the effect of the osteophyte was demonstrated by surgical removal of the osteophyte and resolution of recurrent nerve palsy [5]. Virk et al. and Allensworth et al. also reported cases in which cervical osteophytes caused vocal cord paralysis [6,7]. We found only a few reports of cervical osteophytes presenting with vocal cord paralysis [5-7]. Because the patient was aware of swallowing difficulty even before the injury, the osteophytes may have already caused recurrent nerve damage. The patient's severe head injury with a skull fracture, neck pain, and hematoma in the left posterior neck area was considered to have been accompanied by severe neck flexion and extension movements, which compressed and damaged the surrounding soft tissues, resulting in bilateral vocal cord paralysis.

Clinicians should also be aware of the possibility of delayed vocal cord paralysis, even when strong energy is applied to the neck. As shown in Table 2, more patients had respiratory distress immediately after arrival at the hospital or were intubated due to their level of consciousness, but 40% (4/9) of the patients had respiratory distress on or after the next day of arrival, and 2 of them developed respiratory distress after 10 days. The mechanism of delayed vocal cord paralysis is thought to be ischemia of the recurrent nerve due to inflammation-associated neurotrophic vasoconstriction or nerve damage due to swelling associated with venous stasis [20]. In the present case, the tracheoesophageal groove may have been compressed and damaged by the bone spur that was already present, resulting in delayed vasoconstriction and swelling associated with venous stasis, which may have caused the onset of vocal cord paralysis. At the same time, however, it should be noted that the presence or absence of trauma, such as a fracture or brain injury, cannot determine whether dyspnea is immediate or delayed in onset.

## Conclusions

Bilateral vocal cord paralysis can cause various degrees of dyspnea and is sometimes fatal. In this study, we report a case of delayed bilateral vocal cord paralysis caused by cervical osteophytes due to flexion and extension of the neck following a head injury. Cervical osteophytes can cause recurrent nerve palsy by compressing the tracheoesophageal groove. The present report shows that paralysis can be manifested when combined with trauma, as previously reported. Therefore, it is essential to recognize vocal cord paralysis and dyspnea caused by trauma as a differential diagnosis. It is also important to note that the paralysis may not occur immediately after the injury but may be delayed, as in the present case.
